# Speech Quality Feature Analysis for Classification of Depression and Dementia Patients

**DOI:** 10.3390/s20123599

**Published:** 2020-06-26

**Authors:** Brian Sumali, Yasue Mitsukura, Kuo-ching Liang, Michitaka Yoshimura, Momoko Kitazawa, Akihiro Takamiya, Takanori Fujita, Masaru Mimura, Taishiro Kishimoto

**Affiliations:** 1 Graduate School of Science and Technology, School of Integrated Design Engineering, Keio University, Yokohama 223-8522, Japan; brian.sumali@keio.jp; 2 Department of System Design Engineering, Faculty of Science and Technology, Keio University, Yokohama 223-8522, Japan; mitsukura@keio.jp; 3 Department of Psychiatry, School of Medicine, Keio University, Tokyo 160-8582, Japan; kcliang@keio.jp (K.-c.L.); y-michitaka@keio.jp (M.Y.); m.kitazawa@keio.jp (M.K.); akihiro.takamiya2017@keio.jp (A.T.); mimura@a7.keio.jp (M.M.); 4 Department of Health Policy and Management, School of Medicine, Keio University, Tokyo 160-8582, Japan; tafujita@keio.jp

**Keywords:** pseudodementia, automated mental health screening, audio features, statistical testing, machine learning

## Abstract

Loss of cognitive ability is commonly associated with dementia, a broad category of progressive brain diseases. However, major depressive disorder may also cause temporary deterioration of one’s cognition known as pseudodementia. Differentiating a true dementia and pseudodementia is still difficult even for an experienced clinician and extensive and careful examinations must be performed. Although mental disorders such as depression and dementia have been studied, there is still no solution for shorter and undemanding pseudodementia screening. This study inspects the distribution and statistical characteristics from both dementia patient and depression patient, and compared them. It is found that some acoustic features were shared in both dementia and depression, albeit their correlation was reversed. Statistical significance was also found when comparing the features. Additionally, the possibility of utilizing machine learning for automatic pseudodementia screening was explored. The machine learning part includes feature selection using LASSO algorithm and support vector machine (SVM) with linear kernel as the predictive model with age-matched symptomatic depression patient and dementia patient as the database. High accuracy, sensitivity, and specificity was obtained in both training session and testing session. The resulting model was also tested against other datasets that were not included and still performs considerably well. These results imply that dementia and depression might be both detected and differentiated based on acoustic features alone. Automated screening is also possible based on the high accuracy of machine learning results.

## 1. Introduction

Dementia is a collective symptoms attributed to loss of recent and remote memory along with difficulty in absorbing new knowledge and trouble in decision making. The most common cause of dementia is Alzheimer’s disease which contributes to 60–70% of all dementia cases worldwide. Presently there is no treatment available [1] and recent researches focuses on early detection of dementia signs [2,3,4,5,6,7,8,9] and reducing the risk factors to slow the cognitive decline [10,11,12,13].

Preliminary diagnosis of dementia typically performed in a mental hospital by a licensed psychiatrist interviewing and performing tests to the patients [14,15,16]. Occasionally, diagnosing dementia becomes a complex process, as elderly patients with major depressive disorder often has overlapping symptoms with dementia. To determine whether a patient is truly suffering from dementia, a rigorous test must be performed [17]. A temporary decrease in mental cognition caused by mental disorders is defined as pseudodementia [17,18,19,20,21]. The key difference of pseudodementia is the reversibility of cognitive impairment, in contrast with the progressive nature of dementia. In some cases pseudodementia also serves as biomarker of dementia [21]. Unfortunately, most engineering researches concern only with depression severity or dementia severity [22,23] and almost none focused on pseudodementia.

Features commonly employed for automated mental health screening include facial features (gaze, blink, emotion detection, etc.) [24,25,26], biosignals (electroencephalogram, heart rate, respiration, etc.) [27,28,29,30], and auditory features (intensity, tone, speed of speech, etc.) [23,31]. Although biosignals are the most reliable data source, most of biosignal measurement devices are arduous to equip, limiting their value. In the other hand, facial and acoustic features may be obtained with minimal burden to the patient. As audio feature analysis is comparatively straightforward when compared to facial image analysis, we utilized audio features in this study instead of image features.

The aim of this study was to use an array microphone to record conversations between psychiatrists and depression patients and dementia patients in a clinical setting, and to investigate the differences in acoustic features between the two patient groups and not against healthy volunteers, differing from other conventional studies. Additionally, we are using dataset labelled from licensed psychiatrist to reduce the subjectivity. We revealed the features contributing for pseudodementia screening. In addition, we examined the possibility of utilizing machine learning for automatic pseudodementia screening.

## 2. Materials and Methods

### 2.1. Data Acquisition

This study is conducted as a part of Project for Objective Measures using Computational Psychiatry Technology (PROMPT), a research aimed to develop objective, noninvasive, and easy-to-use biomarkers for assessing the severity of depressive and neurocognitive disorders, including dementia. The details of the project may be found in [32].

**Figure 1:**
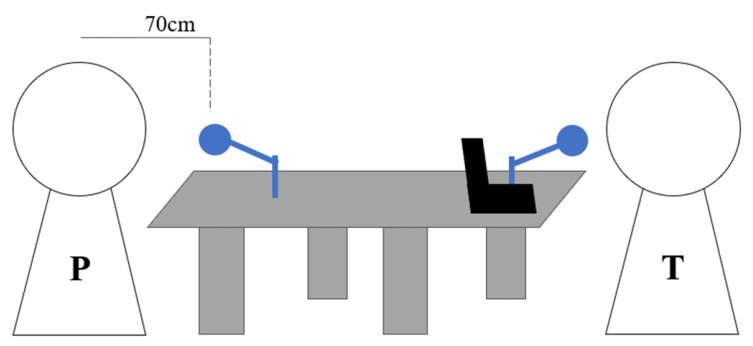
Recording setup during interview session. P is the patient and T is the psychiatrist. There is a distance of approximately 70 cm between the patient’s seat and the recording apparatus.

The PROMPT study was approved by Keio University Hospital Ethics Committee (20160156, 20150427). All participants provided written informed consent. The experiment was conducted on Keio University Hospital and Joint Medical Research Institute. During the interview, the patient and the psychiatrist were seated across a table, as shown in Figure 1.

A single session consists of “free talk” segment followed by “rating” segment. In “free talk”, the psychiatrist conducts a typical clinical interview concerning the patient’s daily life and mood. The length of a “free talk” segment is around 10 min. In the “rating” segment, the patient is interviewed based on a clinical assessment tools related to their mental health history, which may include some tasks such as clock-drawing test and memory test or some personal questions such as their sleep habit and depressive mood in the recent weeks. The duration of “rating” segment typically lasts more than 20 min.

### 2.2. Participants

For statistical analysis, the first, and the second parts of machine learning, several datasets were removed from the PROMPT database in consideration of age features and the presence of symptoms. Only datasets which satisfy the following criteria were included:

1. Age between 57 and 84 years-old; 57 is the lowest age for dementia patients and 84 is the highest age for depression patients. The purpose of this criterion was to remove the effect of age which is positively correlated with Dementia.
2. For dementia patients: mini-mental state examination (MMSE) score of 23 or less accompanied with 15-item geriatric depression scale (GDS) score of 5 or less; The purpose of this criterion was to select only patients with dementia symptoms and exclude patients with both symptoms. A person is defined as symptomatic dementia if the MMSE score is 23 or less.
3. For depression patients: 17-item Hamilton depression rating scale (HAMD17) of 8 or more. A person is defined to be depressed if one’s score of HAMD17 is 8 or more.
4. The recording session was from “free talk” and the length was at least 10 min long. The purpose of this criterion was to ensure enough information contained within the recordings.

For the third part of machine learning, different criteria were applied to PROMPT database to construct a test set consisting of young depressed and old dementia datasets. Specifically, the criteria were:

1. For dementia patients: mini-mental state examination (MMSE) score of 23 or less accompanied with 15-item geriatric depression scale (GDS) score of 5 or less; The age of the patients should be of 85 years or more.
2. For depression patients: 17-item Hamilton depression rating scale (HAMD17) of 8 or more. The age of the patients should be no more than 56 years.
3. The recording session was from “free talk” and the duration was at least 10 min long.

**Figure 2:**
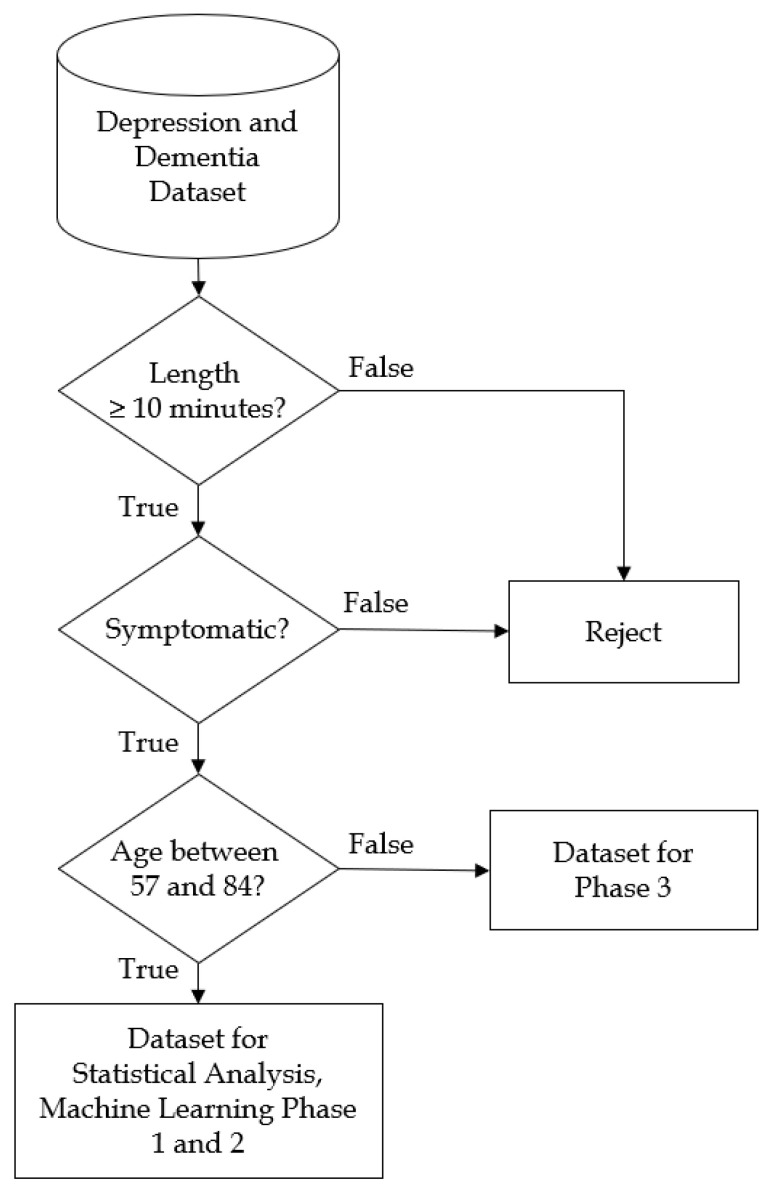
Flowchart of dataset filtration.

Each dataset corresponds to a interview session from one subject. In this study, the datasets were considered as independent because (1) time gap between the sessions were long, 2 weeks in the minimum; and (2) the clinical score results may increase or decrease compared to the first visit, especially depression patients. Figure 2 illustrates the dataset filtering for the statistical analysis and machine learning phases.

### 2.3. Materials

A vertical array microphone: Classis RM30W (Beyerdynamic GmbH & Co. KG, Heilbronn, Germany) with an internal noise cancellation filter to remove wind and pop noise was utilized to record conversations between patients and psychiatrists. The sampling rate was set to 16 kHz. Feature extraction and analysis was performed utilizing typical processor: Dell G7 7588 with Intel Core i7-8750H@2.20 GHz, 16 GB RAM, manufactured in China. with Windows 10 operating system. All methods were available built-in from software MATLAB 2019b.

Clinical assessment tools utilized were 17-item Hamilton depression rating scale (HAMD) [33], 15-item geriatric depression scale (GDS) [16], Young mania rating scale (YMRS) [34] for depression patients; and mini-mental state exam (MMSE) [14] and clinical dementia rating (CDR) [16] for dementia patients. In this study, HAMD for depression and MMSE for dementia is used as golden standard.

### 2.4. Audio Signal Analysis

#### 2.4.1. Preprocessing

In some rare cases, the recordings contained some outliers, possibly caused by random errors, and preprocessing of the raw data needs to be conducted. We defined the outliers by using inter-quartile range (IQR). A point in the audio recording is defined to be an outlier if it satisfies one of the following conditions:

1. X<Q1−1.5IQR
2. X>Q3+1.5IQR

Here, X is the signal, Q1 is the lower (1st) quartile, Q3 is the upper (3rd) quartile, and IQR is the inter-quartile range, computed by subtracting Q1 from Q3. We then apply cubic smoothing spline fitting to the audio signal, without the outliers. The objective of this method is twofold: (1) to interpolate the removed outliers, (2) subtle noise removal.

Additionally, intensity normalization was also performed. This was to ensure that the data is in equal scale to each other and to reduce clipping in audio signals. The normalization was conducted by rescaling the signal such that the maximum absolute value of its amplitude is 0.99. Continuous silence in form of trailing zeroes at front and end of the recordings were also deleted.

#### 2.4.2. Feature Extraction

A subtotal of ten acoustic features were extracted from raw data. They were: Pitch, harmonics-to-noise ratio (HNR), zero-crossing rate (ZCR), Mel-frequency cepstral coefficients (MFCC), Gammatone cepstral coefficients (GTCC), mean frequency, median frequency, signal energy, spectral centroid, and spectral rolloff point, with details in Table 1. These features were chosen as they represent both temporal and spectral features of a signal. Additionally, some of these features relate to closely to speech which is a common biomarker for both depression and dementia [35,36,37]. These features were computed once in every 10 ms by applying a 10 ms window with no overlap. We then performed feature extraction to the windowed signals. The total count of audio feature is 36, with 14 MFCCs and GTCCs. As we used data with length of at least 10 min, a minimum of 60.000 datapoints were obtained, for each feature. We then computed the mean, median, and standard deviation (SD) of the datapoints and used them for statistical analysis and machine learning, resulting in total feature count of 108.

#### 2.4.3. Statistical Analysis

To investigate the relationship between audio features and clinical symptoms, linear correlations of the acoustic features against the corresponding clinical rating tools were computed. The clinical rating tools were HAMD for depression subjects and MMSE for dementia subjects. In addition, two-tailed *t*-test were also performed to check statistical significance. The values were adjusted using Bonferroni correction. Additionally, correlation between age and sex with clinical rating tools were also evaluated for validation purposes.

#### 2.4.4. Machine Learning

Machine learning was performed in three stages: (1) to examine the possibility of automatic pseudodementia diagnosis with unsupervised learning, (2) to examine the possibility of automatic pseudodementia diagnosis with supervised classifier, and (3) to validate its robustness against non age-matched datasets. The unsupervised learning algorithm utilized for the first stage was k-means clustering. The parameters for k-means clustering were k = 2 with squared Eucledian distance metric. For stages 2 and 3, the machine learning model utilized was a binary classifier: support vector machine (SVM) with linear kernel, 3rd order polynomial kernel, and radial-basis function (RBF) kernel [43]. The hyperparameters for both linear kernel and polynomial kernel is the cost parameter C while RBF kernel has two hyperparameters: C and gamma. The optimization of hyperparameters was performed using grid search algorithm with values ranging from $\frac{1}{1000}$ to 1000. Linear kernel was chosen as it allows the visualization of feature contributions, as opposed to SVM with nonlinear kernels. For the second phase, the machine learning session was performed using nested 10-fold cross-validation. It is defined as follows:

1. Split the datasets into ten smaller groups, maintaining the ratio of the classes
2. Perform ten-fold cross validation using these datasets.
  For each fold:
  (a) Split the training group into ten smaller subgroups.
  (b) Perform another ten-fold cross-validation using these subgroups.
    For each inner fold:
    i Perform LASSO regression [44] and obtain the coefficients.
      The LASSO regression solves
      $\min_{\alpha ,\beta }(\frac{1}{2N}\sum_{i=1}^{N}{\left(y_{i}-\alpha -\sum_{j}\beta_{j}x_{ij}\right)}^{2}+\lambda \sum_{j}|\beta_{j}\left|\right)$
      where α is a scalar and β is a vector of coefficients, *N* is the number of observations, $y_{i}$ is the response at observation *i*, $x_{ij}$ is the vector of predictors at observation *i*, and λ is a nonnegative regularization parameter. High value of λ results in stricter feature selection and in this study, it is computed automatically such that it is the largest possible value for nonnull model. The performance of the model is not considered.
    ii Mark the features with coefficient of less than 0.01.
  (c) Perform feature selection by removing features with 10 marks obtained from step 2-b-ii.
  (d) Train an SVM model based on features from (c).
3. Compute the average performance and standard deviation of the models.

**Figure 3:**
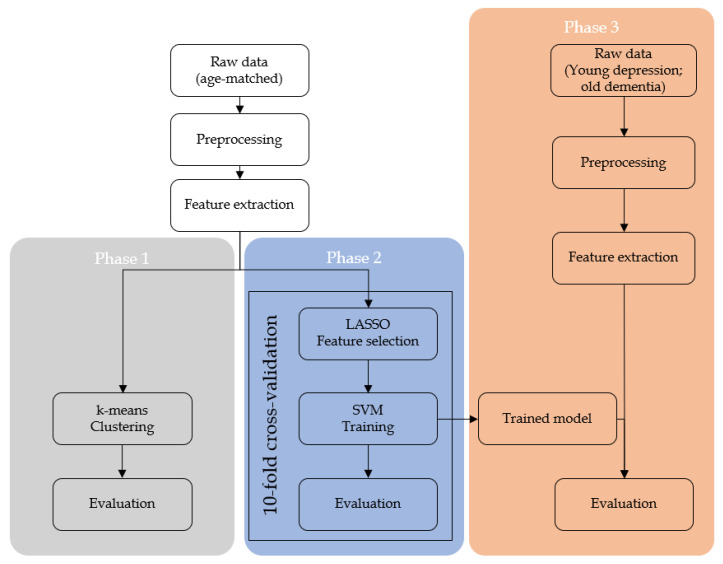
Flowchart of supervised machine learning procedure. The first and second phase used age-matched symptomatic depression and dementia subjects. The first phase consists of unsupervised machine learning clustering while the second phase consists of conventional training and evaluation. The third phase involves of utilizing machine learning model trained from age-matched subjects against non-age matched subjects.

In the third phase, a SVM model was trained using age-matched subjects and selected features from the second phase. Resulting model’s performance is evaluated against the filtered-out subjects: young depression and old dementia subjects. In both cases, the dementia patients were labelled as class 0 (negative) and depression patients were labelled as class 1 (positive). The illustration of the phases are shown in Figure 3.

#### 2.4.5. Evaluation Metrics

**Figure 4:**
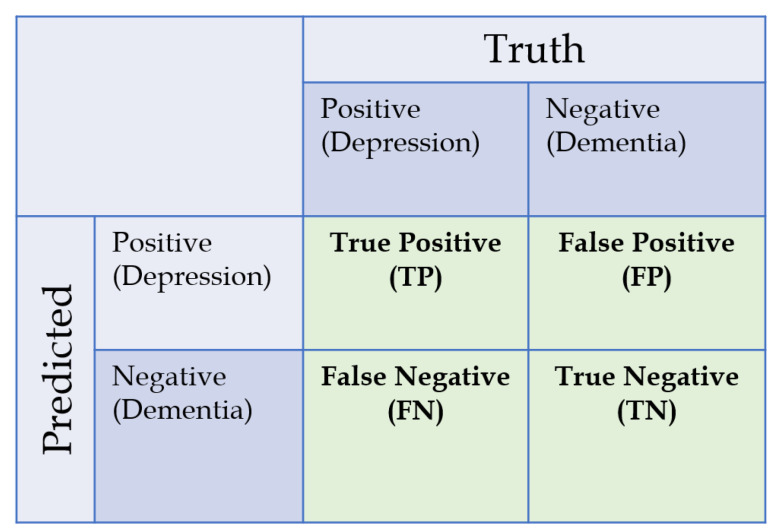
Confusion matrix and class label utilized in this study.

We utilized eight metrices to evaluate the effectiveness of the machine learning model, all of which are computed based on the ratio of true positive (TP), false positive (FP), true negative (TN), and false negatives (FN). In this study, the class depression was labelled as “positive” and dementia was labelled as “negative”. All of the TP, FP, TN, and FN values were obtained from confusion matrix, as shown on Figure 4. Based on the confusion matrices, the evaluation metrices of observed accuracy, true positive rate (TPR/sensitivity), true negative rate (TNR/specificity), positive predictive value (PPV/precision), negative predictive value (NPV), F1-score, Cohen’s kappa, and Matthew’s correlation coefficient (MCC) can be then computed. The formulas for computing these metrics are described in Table 2. These metrics were conventional evaluation metrics utilized in performance evaluation. Metrics related to inter-rater reliability such as Cohen’s kappa and MCC were included to ensure validity of measurement in cases of imbalanced sample problem.

## 3. Results

### 3.1. Demographics

A total of 120 participants (depression n = 77, dementia n = 43) participated in the study, and 419 datasets (300 of depression and 119 of dementia) were obtained. After age-matching, only 177 datasets (89 of depression and 88 of dementia) from 53 participants (depression n = 24, dementia n = 29) were qualified for the first and second phase of machine learning. The test dataset for second phase of machine learning consisted of young depression patients and old dementia patients and was used in the third phase of machine learning. There were 242 datasets (211 of depression and 31 of dementia) from 67 patients (depression n = 53, dementia n = 14). Details of subject demographics were described in Table 3.

### 3.2. Statistical Analysis

**Figure 5:**
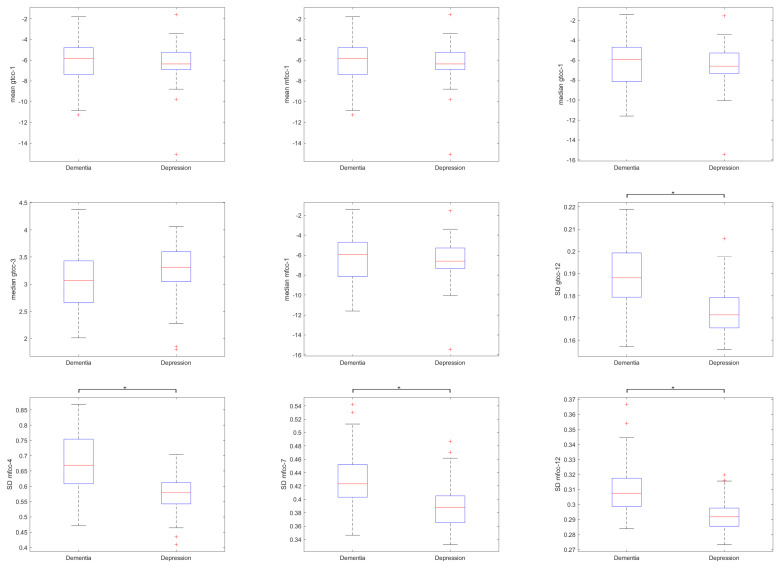
Distribution of features with significant correlation to HAMD and MMSE. * marks the statistically different features between the groups, corrected with Bonferroni correction.

In this section, the statistical analysis for the extracted features were reported. Pearson’s correlation found significant correlations with clinical interview tools in features of GTCCs 1, 3, 12 and MFCCs 1, 3, 4, 7, 12. The average absolute correlation coefficient R was 0.264 and its SD was 0.049. The highest absolute correlation value with statistical significance (*p* < 0.05) was |R|=0.346 for depression and |R|=0.400 for dementia. Features with significant correlation related to depression tend to yield weak to moderate negative Pearson correlation values (average absolute R ± SD = 0.289 ± 0.05) while features with significant correlation related to dementia tend to yield weak to moderate positive Pearson correlation values (average absolute R ± SD = 0.281 ± 0.06). The features’ distributions were depicted in Figure 5 and their corresponding Pearson correlation values were shown in Table 4. Corrected two-tailed *t*-test shows significant differences of features in HNR, ZCR, GTCC coefficients 4–14, mean frequencies, median frequencies, MFCC coefficients 4–13, spectral centroid, and spectral rolloff points. No significant difference was found in Pitch and Energy.

There was no significant correlation was found between sex and clinical assessment tools (depression *R* = 0.021, *p* = 0.853; dementia *R* = 0.142, *p* = 0.928). Age has no significant correlation with depression’s clinical assessment tools (*R* = 0.097, *p* = 0.403) but significant, moderate correlation between age and dementia’s clinical assessment tools was found (*R* = 0.424, *p* = 0.0046).

### 3.3. Machine Learning

In this section, the results of machine learning were presented. The evaluation results from unsupervised learning with kMeans algorithm was shown on Table 5. For the SVM with linear kernels, 26 features were completely rejected in the feature selection, resulting in their removal during creation of the model for second phase. The rejected features were related to pitch, GTCCs 1–3, MFCCs 1–3, signal energy, spectral centroid, and spectral cutoff point. Feature selection in SVM with 3rd order polynomial kernel results in removal of 28 features. The rejected features were related to pitch, GTCCs and MFCCs (1–3, 12–13), signal energy, spectral centroid, and spectral cutoff. LASSO with RBF-SVM similarly rejects 28 features related to low-order (1–4) and high-order (10–13) MFCC and GTCC coeffcients, pitch, signal energy, spectral centroid, and spectral cutoff.

**Figure 6:**
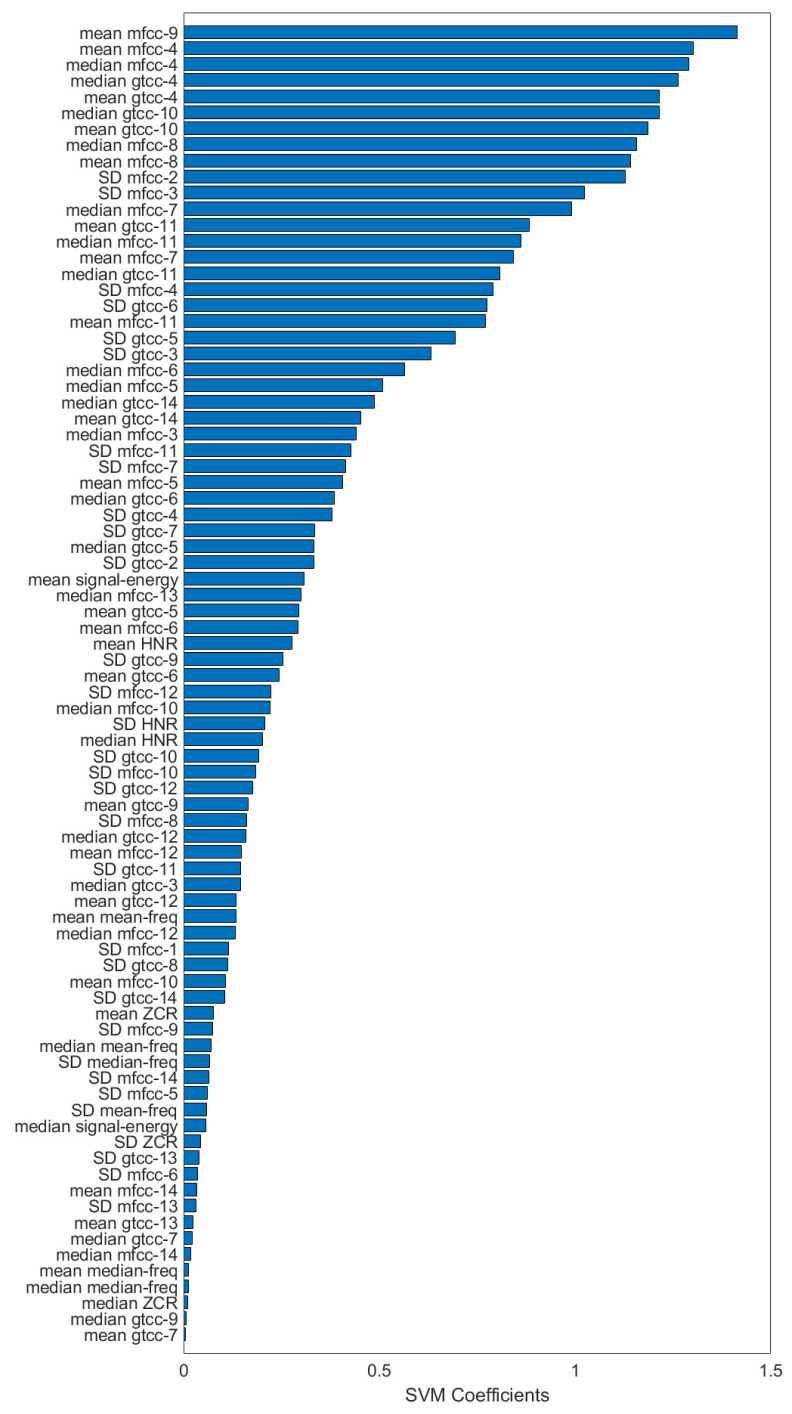
Absolute value of feature contributions of linear SVM with LASSO feature selection, sorted descending.

Results of feature contributions of the trained linear SVM model was presented in Figure 6 alongside with the list of remaining 82 features. The feature contributions were absolute value of linear SVM coefficients. Machine learning evaluation results for phase 2 were shown on Table 6, Table 7 and Table 8 and the results for phase were shown on Table 9. Results with and without LASSO algorithm also shown in these tables to confirm effectiveness of feature selection. Here, the label “positive” represents depression patients and “negative” is for dementia patients.

## 4. Discussion

In the present study, we obtained the audio recordings from clinical interviews of depression and dementia patients. Then, the recordings were filtered according to the analysis criteria. Preprocessing and acoustic feature extraction was then performed to the qualifying datasets. Statistical analysis and machine learning were performed to the acoustic features.

This study has potential limitations. First, although subtle, the recordings were contaminated with the doctor’s voice. This naturally reduces the quality of the acoustic features. Next, there is no removal of silence between the dialogues. We hypothesized that long silences correspond to low motivation and therefore useful for predicting depression. Third, we did not consider real-time appliances. We utilized the full length of the recordings for predicting dementia versus depression. Finally, all the experiments were conducted in Japanese hospital, with Japanese doctors, and with Japanese patient. The speech features we extracted might be specific to the Japanese. Needless to say, these limitations imply potential bias in our study and the results of our study must be interpreted with attention to the limitations.

As a result, we found that GTCC coefficients 1, 3, and 12 along with MFCC coefficients 1, 3, 4, 7, 12 showed significant correlation with both clinical assessment tools: HAMD and MMSE, as shown in Table 4. Interestingly, the sign of Pearson’s correlation coefficient were different; negative correlation was observed for HAMD and positive correlation was observed for MMSE. This suggests that although the features were important for both depression and dementia, they correlated differently. Another thing to note that the highest absolute correlation value with significance (*p* < 0.05) was 0.346 for HAMD and 0.400 for MMSE, suggesting a weak to moderate correlation between the audio features and clinical rating scores.

The corrected *t*-test between these features in Figure 5 showed statistical differences only in certain features. Interestingly, the standard deviation of a rather high-order MFCC coefficient showed significant difference. Normally, most of the information are represented in the lower order coefficients and their distributions were important for speech analysis. Feature contribution shown in Figure 6 puts these features in the middle of the selected features, and some of the lower-order MFCC features were even removed. This might imply the shared features between dementia and depression did not contribute well for predicting them.

Statistical comparison of acoustic features between two groups found significant differences in both temporal and spectral acoustic features. No significant difference between the two groups can be found in pitch and energy, both in the family of temporal features.

Although the result from unsupervised clustering algorithm was not satisfactory, both the accuracy and inter-rater agreement show that the performance was better than chance, denoting the underlying patterns in the data. In the second part of machine learning, feature selection was performed using LASSO algorithm. Here, both pitch and signal energy features were rejected alongside with other spectral features. Considering that both pitch and signal energy also showed no statistical significance in the *t*-test, it can be inferred that these features do not contribute for classification of depression and dementia. In contrast, GTCCs 4–14 and MFCCs 4–14 had statistically significant difference and were also selected by LASSO algorithm. GTCCs and MFCCs are similar features, related to tones of human speech. Although GFCCs was not developed for speech analysis, both are commonly used for speech recognition systems [45,46]. This finding is consistent with the fact that a person’s speech characteristics might be related with their mental health. SVM feature contributions also confirmed that the top contributing features were MFCCs and GTCCs. As the coefficients of the MTCCs and GTCCs are related to the filterbanks utilized when computing them, these coefficients have the benefits of being interpretable [47].

Surprisingly, the best result of the SVM was obtained in SVM with linear kernel, although the the scores were only slightly superior to the nonlinear SVMs. Additionally, the effectiveness of LASSO algorithm for feature selection was evaluated and interesting result was found. For the second phase, all the SVM models benefited from having LASSO feature selection, but for the third phase, nonlinear SVMs seemed to be the most benefited with the feature selection. This might be related by the LASSO algorithm. As LASSO regression is a linear regression with penalty and the feature selection step was basically to discard features that give zero contribution to LASSO regression, linear SVM might be similar to it and was redundant in this case.

Nevertheless, high accuracy and interrater agreement were obtained from the models in both machine learning phases. For comparison, studies [24,25,28,29], and [23] have 87.2%, 81%, 81.23%, 89.71% and 73% as accuracy for predicting depression, respectively. [31] reports 73.6% accuracy for predicting dementia and [30] reports 99.9% TNR and 78.8% TPR. However, most of these studies compared healthy subjects against symptomatic patients, while our study compared patients afflicted with different mental problem. Additionally, most conventional studies measure depression by questionnaire and not with clinical examination, so this cannot be said to be a fair comparison. Low NPV scores and inter-rater during the third phase maybe due to the fact that evaluation in third phase was utilized with heavily imbalanced dataset and with higher number of samples compared to the training phase. These results suggest the possibility of using audio features for automatic pseudodementia screening.

## 5. Conclusions

We recorded the audio of clinical interview session of depression patients and dementia patients in a clinical setting using an array microphone. Statistical analysis shows significant differences in audio features between depressed patients and dementia patients. A machine learning model was constructed and evaluated; considerable performance was recorded for distinguishing depression patients and dementia patients. Feature contribution analysis reveal features MFCC and GTCC features to be the highest contributing features. The top contributing features were 9th and 4th MFCC features. Based on our findings, we conclude that automated pseudodementia screening with machine learning is feasible.

## 6. Future Work

Although this study has yielded considerably good results, there are still some rooms for improvements. For example, to eliminate the psychiatrist’s voice inside the recordings. Although the microphone was situated against the patient, subtle amount of the psychiatrist’s voice also included in the recordings. As such, a specific voice separation algorithm needs to be developed and applied to remove psychiatrist’s voice. This will certainly add silent parts in the recordings and the feature extraction methodology needs to be modified; instead of processing audio with 10 ms window, activity-based window might be considered. Additionally, a dynamic cardioid microphone or multichannel array microphone might be beneficial for picking sounds only from the patient’s side. In this case, room settings for suppressing reverberation and microphone placement becomes very important.

In conjunction with psychiatrist voice removal, activity-based features might also reveal relevance in aspects we did not consider in this study. Here, we hypothesized that longer silence between answers corresponds with lower patient cognition. We assumed that these silence segments will affect the mean value of the features while minimally affecting the median value and is beneficial for differentiating dementia against depression. However, activity-based or content-based analysis might reveal the difference in features we considered irrelevant in this study, such as signal energy.

Also, this study does not consider patients with overlapping symptoms of depression and dementia. Thus, the next step of this study is to develop a multi-class classifier capable of predicting patients with overlapping symptoms. A regression model trained with clinical assessment tools for both depression and dementia is also a possibility.

In consideration of improving the accuracy, more advanced machine learning techniques such as neural network might be suitable. Although the number of available dataset is relatively small for neural networks, sub-sampling and bootstrapping techniques might help to increase the numbers of dataset. Attention must be paid during the validation such that no data leak may occur. Additionally, feature extraction methods such as the combination of numerous hybrid acoustic features, as listed in [48] might also be beneficial. Nevertheless, the curse of dimensionality should be avoided when handling such numerous predictors.

Additionally, while this study did not consider real-time analysis, shorter audio input length should be considered. In this study we used 10 min recording of the “free talk” session and disregarded the processing time. However, in real case, it is more beneficial if the processing was complete before the patient and psychiatrist started the examination with clinical assessment tools.

Finally, in regards the dataset used for training and testing. All experiments were conducted in a Japanese hospital, with Japanese therapist, and with Japanese patient. Although the audio features relating to mental health are supposed to be independent with the language, there is a need to replicate this research outside of Japan and to evaluate the performance of our model against the publicly available databases. Utilizing other databases also have the benefit of the possibility for fair effectiveness evaluation with our model.

## Figures and Tables

**Table 1:** List of features utilized in this study.

Feature	Mathematical Functions and References
Pitch	[38]
Harmonics-to-noise ratio (HNR)	[39]
Zero-Crossing Rate (ZCR)	$ZCR\left(X\right)=\frac{1}{2N}\sum_{i}^{N}\left|sgn\left(X_{i}\right)-sgn\left(X_{i-1}\right)\right|$
Mel-frequency cepstral coefficients (MFCC)	[40]
Gammatone cepstral coefficients (GTCC)	[41]
Mean frequency	Mean of power spectrum from the signal
Median frequency	Median of power spectrum from the signal
Signal energy (*E*)	$E\left(X\right)=\frac{\sigma (X)}{\mu (X)}$
Spectral centroid (*c*)	$c=\frac{\sum_{i=b_{1}}^{b_{2}}f_{i}s_{i}}{\sum_{i=b_{1}}^{b_{2}}s_{i}}$ [42]
Spectral rolloff point (*r*)	$\sum_{i=b_{1}}^{r}s_{i}=\frac{k}{100}\sum_{i=b_{1}}^{b_{2}}s_{i}$ [42]

For ZCR: *N*, sgn, and $X_{i}$ denotes the length of signal, signum function extracting the sign of a real number (positive, negative, or zero), and i-th sequence of signal X, respectively. For mean frequency and median frequency: power spectrum from the signal was applied by performing Fourier transform. For signal energy: E(X) is the signal energy of signal X, σ(X) denotes the function of standard deviation of signal X and μ(X) indicates the function of mean of signal X. For spectral centroid: *c* denotes the spectral centroid, $f_{i}$ is the frequency in Hertz corresponding to bin *i*, $s_{i}$ is the spectral value at bin *i*, and $b_{1}$ and $b_{2}$ are the band edges, in bins, over which to calculate the spectral centroid. For spectral rolloff point: *r* is the spectral rolloff frequency, $s_{i}$ is the spectral value at bin *i*, and $b_{1}$ and $b_{2}$ are the band edges, in bins, over which to calculate the spectral spread.

**Table 2:** List of evaluation metrics.

Metric	Mathematical Formula
Accuracy (ACC)	$ACC=\frac{TP+TN}{TP+TN+FP+FN}$
True positive rate (TPR)	$TPR=\frac{TP}{TP+FN}$
True negative rate (TNR)	$TNR=\frac{TN}{TN+FP}$
Positive predictive value (PPV)	$TPR=\frac{TP}{TP+FP}$
Negative predictive value (NPV)	$TPR=\frac{TN}{TN+FN}$
F1 score	$F1=2\frac{PPV*TPR}{PPV+TPR}$
Cohen’s kappa	$EXP=\frac{\left(TP+FP)(TP+FN)+(TN+FN)(TN+FP\right)}{{TP+TN+FP+FN}^{2}}$
	$Kappa=\frac{ACC-EXP}{1-EXP}$
Matthew’s correlation coefficient (MCC)	$MCC=\frac{TP*TN-FP*FN}{\sqrt{\left(TP+FP)(TP+FN)(TN+FP)(TN+FN\right)}}$

**Table 3:** Subject demographics.

Demographics		Depression	Dementia
Symptomatic	n (dataset/subject)	300/77	119/43
	age (mean ± s.d. years)	50.4 ± 15.1	80.8 ± 8.3
	sex (female %)	54.5	72.1
Age-matched	n (dataset/subject)	89/24	88/29
	age (mean ± s.d. years)	67.8 ± 7.1	77.0 ± 7.5
	sex (female %)	83.3	72.4
Young depression, Old dementia	n (dataset/subject)	211/53	31/14
	age (mean ± s.d. years)	42.5 ± 10.4	88.5 ± 1.9
	sex (female %)	41.5	71.4

**Table 4:** Features with significant Pearson correlation in both depression and dementia patients.

Feature Description	Pearson’s Correlation	
	HAMD Depression	MMSE Dementia
mean GTCC_1	−0.346	0.226
mean MFCC_1	−0.346	0.226
median GTCC_1	−0.325	0.219
median GTCC_3	−0.224	0.230
median MFCC_1	−0.325	0.219
SD GTCC_12	−0.218	0.257
SD MFCC_4	−0.289	0.329
SD MFCC_7	−0.221	0.274
SD MFCC_12	−0.259	0.224

**Table 5:** Phase 1: Unsupervised machine learning result.

Metric	kMeans (%)
Accuracy (ACC)	62.7
True positive rate (TPR)	89.9
True negative rate (TNR)	35.2
Positive predictive value (PPV)	58.3
Negative predictive value (NPV)	77.5
F1 score	70.8
Cohen’s kappa	25.2
Matthew’s correlation coefficient (MCC)	30.0

**Table 6:** Phase 2: Supervised machine learning result—SVM with linear kernel.

Metrices	Training (Mean ± SD %)		Testing (Mean ± SD %)	
	No LASSO	With LASSO	No LASSO	With LASSO
Accuracy (ACC)	90.1 ± 2.4	95.2 ± 0.7	84.2 ± 5.3	93.3 ± 7.7
True positive rate (TPR)	94.4 ± 0.9	98.3 ± 0.9	88.8 ± 10.5	97.8 ± 4.7
True negative rate (TNR)	85.7 ± 4.6	92.6 ± 1.2	79.6 ± 11.5	89.4 ± 13.7
Positive predictive value (PPV)	87.1 ± 3.5	92.1 ± 1.2	82.5 ± 8.8	90.4 ± 11.7
Negative predictive value (NPV)	93.8 ± 1.0	98.4 ± 0.8	88.8 ± 8.9	98.0 ± 4.2
F1 score	90.6 ± 2.0	95.1 ± 0.7	84.8 ± 5.5	93.5 ± 7.2
Cohen’s kappa	80.2 ± 4.7	90.5 ± 1.4	68.3 ± 10.5	86.7 ± 15.0
Matthew’s correlation coefficient (MCC)	80.5 ± 4.4	90.6 ± 1.4	69.8 ± 10.3	87.8 ± 13.5

**Table 7:** Phase 2: Supervised machine learning result—SVM with 3rd order Polynomial kernel.

Metrices	Training (Mean ± SD %)		Testing (Mean ± SD %)	
	No LASSO	With LASSO	No LASSO	With LASSO
Accuracy (ACC)	91.5 ± 3.1	94.6 ± 8.1	79.1 ± 7.6	89.7 ± 11.4
True positive rate (TPR)	96.4 ± 2.4	99.1 ± 1.0	85.3 ± 10.8	96.7 ± 5.4
True negative rate (TNR)	86.5 ± 4.0	90.0 ± 16.1	72.6 ± 14.3	83.1 ± 22.9
Positive predictive value (PPV)	87.9 ± 3.5	92.3 ± 9.9	76.9 ± 8.3	87.6 ± 13.8
Negative predictive value (NPV)	95.9 ± 2.7	98.9 ± 1.2	84.1 ± 9.9	96.9 ± 5.0
F1 score	91.9 ± 2.9	95.3 ± 6.1	80.3 ± 6.9	91.1 ± 8.2
Cohen’s kappa	82.9 ± 6.3	89.2 ± 16.2	58.0 ± 15.2	79.7 ± 21.7
Matthew’s correlation coefficient (MCC)	83.3 ± 6.2	90.1 ± 13.7	59.4 ± 14.6	81.8 ± 17.9

**Table 8:** Phase 2: Supervised machine learning result—SVM with RBF kernel.

Metrices	Training (Mean ± SD %)		Testing (Mean ± SD %)	
	No LASSO	With LASSO	No LASSO	With LASSO
Accuracy (ACC)	90.4 ± 6.2	95.6 ± 1.9	75.3 ± 12.4	88.7 ± 7.9
True positive rate (TPR)	96.4 ± 2.9	98.8 ± 1.0	77.5 ± 16.6	91.0 ± 10.3
True negative rate (TNR)	84.3 ± 10.2	92.4 ± 3.0	72.9 ± 17.3	86.1 ± 13.1
Positive predictive value (PPV)	86.7 ± 7.9	93.0 ± 2.6	75.6 ± 13.8	88.3 ± 10.4
Negative predictive value (NPV)	95.7 ± 3.7	98.6 ± 1.2	77.6 ± 14.6	91.3 ± 8.9
F1 score	91.2 ± 5.4	95.8 ± 1.7	75.7 ± 12.5	89.1 ± 7.9
Cohen’s kappa	80.8 ± 12.3	91.2 ± 3.7	50.5 ± 24.8	77.3 ± 15.9
Matthew’s correlation coefficient (MCC)	81.5 ± 11.7	91.4 ± 3.6	51.8 ± 25.0	78.3 ± 15.4

**Table 9:** Phase 3: Machine learning result against non-age matched dataset.

Metrics	Linear		Polynomial		RBF	
	All Feats	LASSO	All Feats	LASSO	All Feats	LASSO
Accuracy (ACC)	83.5	82.6	80.2	81.4	65.7	81.0
True positive rate (TPR)	87.7	83.9	82.5	82.9	66.8	82.9
True negative rate (TNR)	54.8	74.2	64.5	71.0	58.1	67.7
Positive predictive value (PPV)	93.0	95.7	94.1	95.1	91.6	94.6
Negative predictive value (NPV)	39.5	40.4	35.1	37.9	20.5	36.8
F1 score	90.2	89.4	87.9	88.6	77.3	88.4
Cohen’s kappa	36.5	42.8	34.6	39.3	13.9	37.3
Matthew’s correlation coefficient (MCC)	37.2	45.7	37.0	42.2	17.3	39.9
